# Dual Function of Histone H3 Lysine 36 Methyltransferase ASH1 in Regulation of Hox Gene Expression

**DOI:** 10.1371/journal.pone.0028171

**Published:** 2011-11-28

**Authors:** Yujiro Tanaka, Koji Kawahashi, Zen-Ichiro Katagiri, Yasuhiro Nakayama, Milind Mahajan, Dimitris Kioussis

**Affiliations:** 1 Genome Structure and Regulation, School of Biomedical Science and Biochemical Genetics, Medical Research Institute, Tokyo Medical and Dental University, Tokyo, Japan; 2 Department of Genetics, Yale University School of Medicine , New Haven, Connecticut, United States of America; 3 Yale Center for Genome Analysis, Yale University, Orange Connecticut, United States of America; 4 Molecular Immunology, National Institute for Medical Research, London, United Kingdom; Ludwig-Maximilians-Universität München, Germany

## Abstract

Hox genes play important roles in haematopoietic development in mammals. ASH1 is a member of the trithorax group (trxG) that is required for proper expression of Hox genes and is preferentially expressed in haematopoietic stem cells. We have recently reported that ASH1 methylates histone H3 at lysine 36 (K36) but its biological function has remained elusive. Here we show that ASH1 regulates Hox gene expression positively and negatively in a leukemic cell line K562 and is required for myelomonocytic differentiation of murine haematopoietic stem cells. ASH1 binds to endogenous Hox loci in K562 cells and its knockdown causes reduced expression of Hox genes. In addition, ASH1 and MLL1 induce more than 100-fold activation of Hox promoters in HeLa cells if expressed simultaneously but not individually. Notably, ASH1 harbouring a point mutation that kills methyltransferase activity is more efficient than wild type ASH1 in Hox gene activation, indicating that K36 methylation is not a prerequisite for Hox gene expression. Moreover, tethering wild type or catalytically inactive methyltransferase domain of ASH1 to a heterologous promoter causes downregulation or upregulation, respectively, of transcription, supporting a hypothesis that K36 methylation imparts repression. Knockdown of ASH1 in K562 cells *in vitro* causes increased expression of ε-globin gene and reduced expression of myelomonocytic markers GPIIb and GPIIIa, whereas knockdown of ASH1 in murine haematopoietic stem cells *in vivo* results in decreased number of macrophages and granulocytes, a phenotype similar to that induced by loss of *mll1* function. Taken together, our data suggest that ASH1 and MLL1 synergize in activation of Hox genes and thereby regulate development of myelomonocytic lineages from haematopoietic stem cells.

## Introduction

Hox genes are arranged in tandem arrays in the genome, and their spacio-temporal expression patterns are regulated by antagonistic functions of Polycomb-group (PcG) and trithorax-group (trxG) proteins which define expression domains of Hox genes in the genome as well as along the body axis[1]–[3]. Loss of either PcG or trxG results in misexpression or loss of expression of Hox genes, respectively, resulting in homeotic transformations of body segments. ASH1 was discovered by screens of imaginal disc mutants in *Drosophila melanogaster* [4]. ASH1 is a member of trxG and loss of its function causes anterior transformations similar to those in *trx* mutants[5]. Trans-heterozygous *ash1/trx* mutants exhibit more severe phenotype at higher penetrance than single mutants, providing genetic evidence for interaction between ASH1 and TRX in Hox gene regulation[6].

We have previously reported that ASH1 methylates histone H3 selectively at K36[7], which has been confirmed by two independent studies of other laboratories[8], [9]. There is also genetic evidence for antagonism between ASH1 and K36 demethylase dKDM2 in *Drosophila* [10], strongly supporting the specificity of ASH1 for K36. TRX and its mammalian homologues MLL1-4 are known to methylate histone H3 lysine 4 (K4)[11], [12]. Precisely how trxG regulates activities of RNA polymerase II remains elusive. The observation that loss of both PcG and trxG restores expression of Hox genes in normally expressed domains implies that trxG is not essential for Hox gene expression but functions primarily by counteracting repressive effects of PcG[13]. Lending support for such a hypothesis, K36 methylation has been recently shown to directly interfere with K27 methylation by PRC2 of PcG[8], [14]. An alternative mechanism of counteracting repressive effects of PcG might be removal of K27 methylation marks by demethylase enzymes contained in MLL family complexes[15], [16]. Reciprocally, K36 demethylase dKDM2 constitutes a PcG subcomplex of dRING[10], suggesting that PcG might antagonize trxG by removing K36 methylation marks. These findings collectively suggest that Hox genes are regulated by dynamic balance between mutually exclusive methylation status of K36 and K27 through competing actions of methyltransferases and demethylases. However, there has been no evidence showing that K36 methylation contributes to Hox gene activation by inhibiting K27 methylation.

Recently, trxG complexes have been shown to contain an essential elongation factor P-TEFb, raising a possibility that trxG might directly affect the rate limiting step of RNA synthesis[17]. Thus, RNA polymerase complexes are “paused” at immediate downstream of transcription initiation site in many developmentally regulated genes[18], and CDK9 activates transcription by phosphorylating and inducing dissociation of NELF/DSIF that anchor RNA polymerase II[19], [20]. The N-terminal part of mammalian ASH1 is most similar to SETDB1[7], [21]. Notably, both SETDB1[22] and MLL1[23] are known to interact with SET oncoprotein and protein phosphatase 2A (PP2A), latter of which could negatively regulate P-TEFb[24]. It thus appears that trxG contains/interacts with both the molecular switch of transcription (P-TEFb) and its regulators (SET/PP2A).

Hox genes play important roles in haematopoietic development[25] and mutations of the *mll1* gene is the major cause of infant leukaemia[26], [27]. Expression of MLL-AF9 translocation products in murine myeloid precursor cells has been shown to induce leukaemia in association with altered expression patterns of “stem cell genes”[28], [29]. Therefore, understanding molecular mechanisms of how trxG proteins regulate target genes in haematopoiesis has clinical implications for combating leukaemia. ASH1 is preferentially expressed in haematopoietic stem cells in the bone marrow[30] and undifferentiated precursors of T cells in the thymus[31], suggesting that ASH1 might play a role in haematopoietic development. In the current study, a full-length ASH1 expression vector was used to directly assess biochemical function of ASH1 in Hox gene activation *in vitro*. We show that ASH1 synergizes strongly with MLL1 to activate Hox genes. Contrary to expectations, however, our data indicate that K36 methyltransferase activity of ASH1 causes repression rather than activation of Hox genes. We also show that function of ASH1 and MLL1 is required for normal development of myelomonocytic lineages from murine haematopoietic stem cells *in vivo*.

## Results

### ASH1 is required for endogenous Hox gene expression

**Figure 1:**
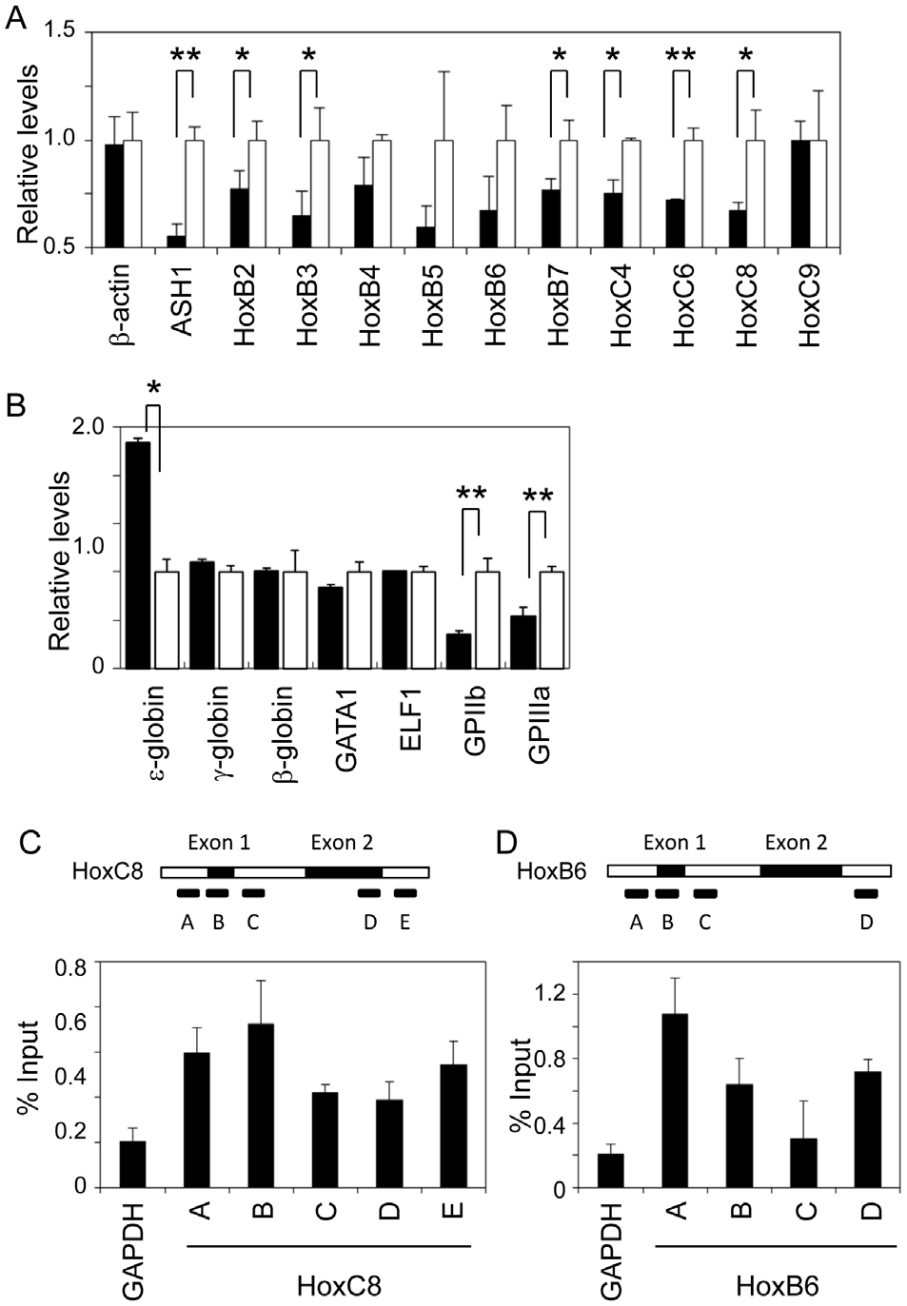
Regulation of endogenous Hox genes by ASH1. (A) Quantitative RT-PCR analysis of HoxB and HoxC gene expression in K562 cells. Expression levels are normalized against average of K562 cells transfected with scrambled oligonucleotides. ASH1 knockdown oligonucleotides (si6925) causes reduction of ASH1 and several HoxB and HoxC genes, whereas β-actin is not affected. Values are average of triplicates of K562 samples and error bars denote standard deviation. (B) Effects of ASH1 knockdown on erythroid and monocytic marker gene expression in K562 cells. ASH1 knockdown causes increased expression of the ε-globin gene and decreased expression of GPIIb and GPIIIa genes. Transcription factors required for globin gene expression such as GATA1 and ELF1 are not affected. Data represent triplicate K562 samples and error bars indicate standard deviation. (C–D) Chromatin immunoprecipitation analysis of ASH1 in the HoxC8 (C) and HoxB6 (D) genes of K562 cells using antibodies against an internal epitope of ASH1. Quantitative PCR signals are shown by proportion to those of input DNA. GAPDH promoter is a negative control to which ASH1 does not bind. ASH1 is associated with promoter and coding regions of HoxC8 and HoxB6 genes. Values are average of triplicates of independent ChIP samples and indicate semi quantitative PCR signals relative to input DNA. Statistically significant differences (Student's t-test) are indicated by asterisks (*: p-value < 0.05, **: p-value < 0.01).

To assess the role of ASH1 in regulation of endogenous Hox gene expression, we analysed the impact of ASH1 knockdown on a human erythroleukaemia cell line K562[32]. To this end, K562 cells were transfected with either siRNA for ASH1 (si6925) or a control scrambled sequence and levels of endogenous Hox genes were quantitated by RT-PCR. Hox genes in Hox-B and Hox-C clusters were analysed because these are expressed in K562 cells as judged by the presence of both RNA polymerase II and active chromatin marks such as K4me3 and K36me3 (Fig. S1). As illustrated in Fig. 1A, si6925 reduced the level of ASH1 to approximately half of the control. Notably, si6925 caused down-regulation of Hox-B2, B3, B5, B6, B7, C4, C6, and C8 to various degrees. These effects were marginal probably because of incomplete depletion of ASH1 by si6925, which might also explain why HoxC9 was not affected by ASH1 knockdown since HoxC9 appears to be transcribed more actively than other genes in the HoxC cluster as judged by H3K4 methylation (Fig. S1). Nevertheless, β-actin (Fig. 1A), γ- and β-globin genes, GATA1, and KLF1 (Fig. 1B) were not affected by si6925, indicating that the effects are gene specific. In addition, expression of both GPIIb and GPIIIa were reduced by ASH1 knockdown (Fig. 1B), supporting the consistency of the effect of ASH1 knockdown. Therefore, these data suggest that ASH1 is required at least partially for expression of HoxB and HoxC genes in K562 cells.

Next, to determine if ASH1 directly regulates Hox gene expression, binding of ASH1 proteins to endogenous Hox loci was assessed by chromatin immunoprecipitation analysis. The HoxC8 gene was first examined because it is expressed in K562 cells, is affected by ASH1 knockdown, and has been also shown to be regulated by MLL1[12], [33]. Fig. 1C and 1D shows that ASH1 is associated with both promoter-proximal regions near the transcription start site and downstream regions of the HoxC8 and HoxB6 genes. These data were obtained by using anti-ASH1 antibodies specific for an internal epitope. Similar experiments using antibodies against the C-terminal end of ASH1 showed less pronounced but similar binding patters of ASH1 to the promoter and downstream region of the HoxC8 gene (Fig. S2). These data are consistent with a recent study showing that ASH1 binds to extended regions of actively transcribed Hox genes in *Drosophila* [34] and support a hypothesis that ASH1 directly regulates transcription of Hox genes through interaction with both promoter and downstream regions of the genes.

### ASH1 and MLL1 co-operates in Hox promoter activation *in vitro*

**Figure 2:**
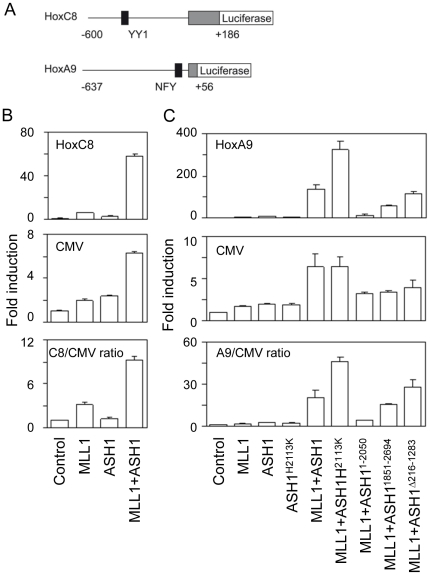
Activation of Hox promoters by ASH1 and MLL1. (A) Luciferase reporter constructs harbouring human HoxC8 (−600 to +186) and HoxA9 (−637 to +56) promoters. (B) Activation of HoxC8-firefly luciferase and CMV-renilla luciferase reporters by MLL1, ASH1, or both together in HeLa cells. HoxC8-specific effects are shown by C8/CMV ratios. Values are average of triplicates of independently transfected HeLa cells and error bars denote standard deviation. (C) Similar to (B) but using HoxA9-firefly luciferase vector and ASH1 mutants including catalytically inactive ASH1 (H2113K) which induces twice as strong activation of HoxA9 (but not CMV) promoter as wild type ASH1. ASH1^1851-2694^ that consists of the C-terminal region has significant activity towards HoxA9. ASH1^Δ216-1283^ lacking a part of the N-terminal region can be detected by Western blot and has the same transactivation potential as wild type ASH1.

ASH1 and TRX belong to trxG and have been shown to genetically interact with each other in *Drosophila* [5], [6]. In mammals, MLL1 has been shown to be required for Hox gene expression[35], and introduction of full-length MLL1 into *mll1*-deficient murine fibroblasts restores expression of HoxC8 and HoxA9 genes[12]. MLL1 is also known to associate with HoxA9 promoter in HeLa cells[11]. To examine if ASH1 and MLL1 interact with each other in mammalian cells, we used luciferase reporter constructs harbouring HoxC8 and HoxA9 promoters (Fig. 2A). HeLa cells which express minimal levels of Hox genes were transiently transfected with Hox promoter-renilla luciferase reporter plasmids together with CMV-firefly luciferase reporter and expression vectors of either MLL1 or ASH1, or both. As illustrated in Fig. 2B (HoxC8) and 2C (HoxA9), expression of MLL1 or ASH1 alone had little impact on Hox and control CMV promoters. By contrast, co-expression of MLL1 and ASH1 caused marked activation of HoxC8 and HoxA9 promoters by 50 to 150-fold (Table S2). The CMV promoter was also activated by several-fold but the effects were 10 to 20-fold more pronounced for Hox promoters (C8 or A9/CMV ratios in Fig. 2C). These effects were also dose-dependent since increasing amount of ASH1 or MLL1 in the presence of fixed amount of MLL1 or ASH1, respectively, caused stronger activation of Hox promoters (Fig. S3).

Despite the clear evidence that co-transfection of ASH1 and MLL1 expression vectors causes strong activation of Hox promoters, ASH1 proteins of a predicted molecular weight failed to be detected by Western blot analysis using antibodies against ASH1 (C-terminal end or internal) or the N-terminal FLAG tag. We therefore constructed several deletion mutants of ASH1 and found that ASH1^Δ216-1283^ lacking a part of the N-terminal SETBP1 homology domain of ASH1 could be detected by Western blot (Fig. S4B) and possessed a Hox promoter activation potential as good as wild type ASH1 (Fig. 2C). The C-terminal part of ASH1, i.e. ASH1^1851-2964^ which harbours all the chromatin-associated motifs but lacks the SETBP1 homology domain, could be detected by Western blot (Fig. S4B) and caused activation of the HoxA9 promoter. Thus, the C-terminal part of ASH1 is largely sufficient to co-operate with MLL1 to regulate Hox gene expression.

### K36 methylation by ASH1 has a gene repressive effect

A point mutation in the SET domain of TRX in *Drosophila* prevents methylation of histone H3 K4 and causes homeotic phenotypes[36]. In addition, mutation of ASH1 in flies results in reduction of histone H3 K4 methylation[37], suggesting that ASH1 plays an important role in regulation of the methyltransferase activity of TRX. However, it remains to be shown if methylation of K36 by ASH1 is required for Hox gene activation. To address this issue, we used an expression vector of ASH1 carrying a point mutation in the SET domain (replacement of histidine^2113^ with lysine inside the coenzyme binding pocket) that kills the methyltransferase activity[7]. Surprisingly, co-expression of MLL1 and the catalytically inactive ASH1^H2113K^ induced twice as strong activation of HoxA9 as wild type ASH1 as shown in Fig. 2B. By contrast, ASH1^H2113K^ alone had little impact on Hox promoters, suggesting that the mutant ASH1 is still functionally dependent on MLL1.

**Figure 3:**
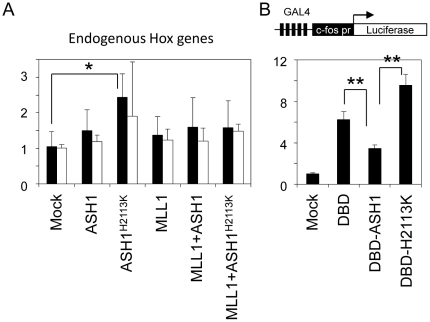
Function of methyltransferase-deficient ASH1. (A) Effect of ASH1 and its mutants on endogenous HoxC8 (closed bars) and HoxB6 (open bars) genes in K562 cells. Catalytically inactive ASH1^H2113K^ augments endogenous Hox genes. Data represent triplicate K562 samples and error bars indicate standard deviation. Endogenous HoxC8 activation by ASH1^H2113K^ is statistically significant (*: p-value < 0.05). (B) Top: a reporter construct harbouring GAL4 binding sequences (x4) and c-fos promoter. Bottom: Expression vectors carrying GAL4-DNA binding domain (DBD), DBD fused to wild type ASH1 SET domain (DBD-ASH1), or DBD fused to catalytically inactive ASH1 SET domain (DBD-H2113K) were co-transfected with the reporter into HeLa cells. Wild type ASH1 SET domain is approximately 3-fold less efficient than catalytically inactive ASH1 SET domain, suggesting that K36 methylation attenuates transcription. Data represent triplicate HeLa samples and error bars indicate standard deviation. Differences between DBD and DBD-ASH1 and between DBD-ASH1 and DBD-H2113K were statistically significant (*: p-value < 0.01).

The apparent lack of requirement of K36 methylation for Hox gene activation could be due to an artificial condition of using episomal targets that may not be organized in proper chromatin structure[38]. To test such a possibility, we next analysed the effect of ASH1^H2113K^ on endogenous Hox genes. As shown in Fig. 3A, expression of ASH1^H2113K^ alone in K562 cells augmented expression of endogenous HoxC8 gene approximately by 2.5-fold. By contrast, wild type ASH1 had little effect on endogenous Hox genes. These data suggest that methyltransferase-deficient ASH1^H2113K^ is more efficient than wild type ASH1 in Hox gene activation, clearly indicating that K36 methylation is not prerequisite for Hox gene activation contrary to a hypothesis that ASH1 might activate Hox genes by methylating K36 and thereby interfering with K27 methylation by PRC2[8].

### Tethering of catalytically inactive ASH1 to a heterologous promoter causes gene repression

5Neither ASH1^H2113K^ nor wild type ASH1 affected endogenous Hox genes when co-transfected with MLL1 (Fig. 3A). This would be unexpected if hyper-activation of Hox genes by ASH1^H2113K^ were mediated simply by increased amount of ASH1 and MLL1 proteins binding to more Hox targets. A possible explanation may be that exogenous ASH1^H2113K^ replaced endogenous ASH1 that is already bound to Hox targets and caused reduction of K36 methylation and consequently derepression of Hox genes. To clarify this issue, we directly tested if ASH1-mediated K36 methylation has a gene repressive effect by artificially tethering its SET domain to the c-fos promoter through a GAL4-DNA-binding domain (DBD). As illustrated in Fig. 3B, the level of reporter gene expression was lower in cells transfected with wild type ASH1 SET domain (DBD-ASH1) than in cells transfected with DBD alone, suggesting that the wild type ASH1 SET domain has a gene repressive effect. By contrast, transfection of DBD-H2113K harbouring the catalytically inactive ASH1 SET domain induced higher levels of reporter gene expression than control GAL4-DBD, suggesting that the ASH1 SET domain has an intrinsic ability to activate transcription above background levels. Compared to DBD-H2113K, DBD-ASH1 attenuates reporter gene expression by approximately three-fold, which is comparable to gene repression observed by fusing GAL4-DBD to Symd2 that is a K36 methyltransferase[39].

### ASH1 regulates myelomonocytic development

K562 cells transfected with ASH1 knockdown oligonucleotides turned light pink in colour (implying haemoglobin synthesis), prompting us to further analyse expression of lineage markers. As illustrated in Fig. 1B, knockdown of ASH1 caused upregulation of the ε-globin gene and downregulation of myelomonocytic markers GPIIb and GPIIIa, suggesting that ASH1 may be involved in erythroid vs. myelomonocytic cell fate determination. K562 cells are derived from blast crisis of chronic myelogenous leukaemia patient[40] and has been shown to undergo erythroid differentiation upon various stimulations such as haemin and chemotherapeutic agents[32], [41]. Our observation that ε- but not γ- or β-globin genes was affected by ASH1 knockdown is consistent with previous reports showing that K562 cells synthesize only foetal type globins[32]. Quantitation of globin gene transcription factors such as GATA1 and KLF1 revealed no changes upon ASH1 knockdown (Fig. 1B), implying a distinct mechanism for regulation of globin genes.

**Figure 4:**
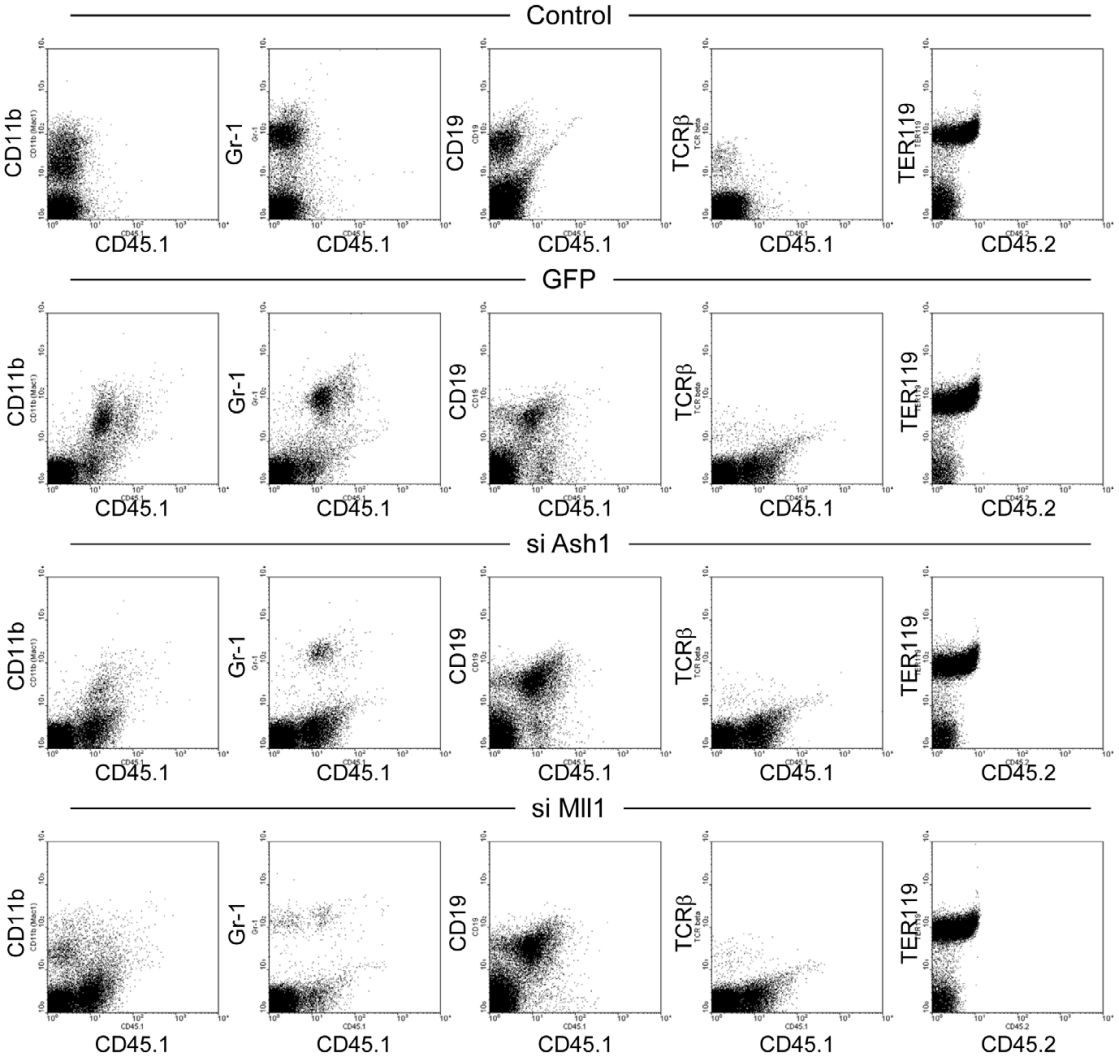
Effect of ASH1 knockdown on haematopoiesis. Representative FACS plots of peripheral blood from mice transplanted with control GFP or knockdown vectors for ASH1 or MLL1. Horizontal axis represent CD45.1 (donor allotype) or CD45.2 (recipient allotype) and vertical axis indicates lineage markers: CD11b (macrophages), Gr-1 (granulocytes), CD19 (B cells), TCRβ (T cells), and TER119 (erythrocytes).

The effect of ASH1 knockdown on gene expression in K562 cells could just reflect the partially committed nature of K562 cell line. To further assess the role of ASH1 in normal haematopoiesis *in vivo*, lineage marker-negative haematopoietic progenitor cells were isolated from the bone marrow of C57BL/6J-Ly5.1 mice, transduced with lentiviral vectors expressing either control GFP or siRNA for ASH1 or MLL1, and then transferred into sublethally irradiated C57BL/6J mice (Ly5.2 allotype). Commitment of donor-derived progenitor cells into different lineages was assessed by flow cytometric analysis of peripheral blood using antibodies against Cd11b (macrophages), Gr-1 (granulocytes), CD19 (lymphocytes), TCRβ (T cells), and TER119 (erythrocytes). As shown in Fig. S6, there is a clear tendency that there are fewer CD11b^+^ and Gr-1^+^ cells and more CD19^+^ cells among donor-derived Ly5.1 (CD45.1)^+^ cells expressing siRNA for ASH1 or MLL1 than in those expressing control GFP. Representative FACS profiles as depicted in Fig. 4 shows that knockdown of ASH1 and MLL1 in haematopoietic progenitor cells skews differentiation from myelomonocytic (CD45.1^+^CD11b^+^ and CD45.1^+^Gr-1^+^ cells) towards lymphoid (CD45.1^+^CD19^+^ cells) or erythroid (CD45.2^-^TER119^+^ cells) lineages as compared to progenitor cells expressing control GFP. The effects of MLL1 knockdown are highly consistent with previous studies showing that *mll1* knockout in mice causes reduction in the number of myeloid and macrophage colonies in culture of yolk sac and bone marrow cells[42], [43]. Taken together, these data suggest that ASH1 functions in the same genetic pathway as MLL1 to facilitate myelomonocytic differentiation of haematopoietic stem cells.

## Discussion

ASH1 is known to interact with trxG and PcG genes in regulation of Hox gene expression[5], [6], [13], but the molecular mechanism of how ASH1 regulates Hox genes has been largely unknown. The current study using reporters carrying HoxC8 and HoxA9 promoters demonstrated that mammalian ASH1 and MLL1 synergize strongly in transcriptional activation of Hox promoters, providing a basis for biochemical analysis of transcriptional regulation. Using such a system, we provided evidence that the methyltransferase activity of ASH1, and K36 methylation by inference, is not required for Hox gene activation but rather imparts repression. Of note, recent studies have revealed that K36 methylation interferes with K27 methylation by PRC2[8], [14] providing a possible mechanism of how ASH1 might counteract repressive effects of PcG[13]. Importantly, however, functional consequences of K36 methylation were not directly addressed in these studies. Our data clearly indicate that K36 methylation is not the major mechanism of how ASH1 activates targets, given that catalytically inactive ASH1 was more efficient than wild type ASH1 in Hox gene activation (Fig. 2C). Taken together, these observations might imply that K36 methylation represents a state of repression distinct from K27 methylation. ASH1 may antagonize PcG repression by preventing “deeper” silencing by K27 methylation.

In *ash1* mutant flies, *Antennapedia (Antp)* promoter-reporter gene has been found ectopically expressed in larvae along the anterior margin of haltare discs where *Antp* is not normally expressed[44], suggesting that ASH1 suppresses *Antp* outside normally expressed domains. Consistent with such a notion, we have reported that expression of the protein-protein interaction domain of ASH1 (i.e. PHD finger) causes increased expression of Hox genes presumably by a dominant negative effect[31]. MLL1 is also known to interact with a transcriptional repressor Sin3A[11], suggesting that trxG could have gene repressive function. In yeast, co-transcriptional methylation of K36 confers memories of recent transcription to cells and represses spurious intragenic transcription initiation through recruitment of Rpd3[45]. Our finding that K36 methylation by ASH1 causes gene repression is consistent with these studies and underscores a notion that trxG proteins have dual function in Hox gene activation.

Recent findings that TRX/MLL-family proteins interact with P-TEFb imply that trxG complexes might regulate transcription by releasing “paused” RNA polymerase II[17]. Activities of P-TEFb is thought to be regulated by sequestration into inactive compartment by 7SK snRNP and HEXIM proteins, and dephosphorylation by protein phosphatase 1 and PP2A[46]. Although it remains to be shown how trxG complexes regulate the activity of P-TEFb, it is of note that MLL1 interacts with PP2A through oncoprotein SET [23] and that the N-terminal part of ASH1 is most similar to SETBP1 that also interacts with oncoprotein SET[7], [22]. SET oncoproteins are known to inhibit the activity of PP2A and thus are expected to activate P-TEFb. Consistent with such a hypothesis, we found that okadaic acid enhances activation of the HoxA9 promoter (but not the CMV promoter) in the presence of ASH1 and MLL1 at a concentration that blocks PP2A (Fig. S5). However, C-terminal region of ASH1 lacking the SETBP1 homology domain has a significant Hox promoter activation potential (Fig. 2C), suggesting that there is yet unknown function of ASH1 for activation of Hox genes.

Our *in vivo* knockdown experiments show that ASH1 and MLL1 play important roles in myelomonocytic development, consistent with previous studies showing that targeting the *mll1* gene causes severe defects in myelomonocytic development in yolk sac and bone marrow[42], [43]. By contrast, more recent studies on conditional knockout of *mll1* demonstrated that MLL1 is not required for postnatal steady-state haematopoiesis but plays an important role in maintenance of stem cell pools in a quiescent state[47], [48]. The cause of such discrepancy is unknown but a possible explanation may be difference in timing and cell type specificity of transgenic Cre-mediated conditional deletion of *mll1* gene and lentivirus-mediated knockdown of *mll1*. Nevertheless, under the same experimental conditions in our system, depletion of ASH1 and MLL1 caused similar phenotypes reinforcing the idea that ASH1 and MLL1 synergize in regulation of haematopoietic development. Further studies on target genes of ASH1 and MLL1 might reveal precise molecular functions of these epigenetic regulators in normal haematopoietic development as well as in leukaemias caused by mutations of *mll1* [28].

## Materials and Methods

### Ethics statement

All animal works were approved and conducted according to the guidelines of Committees of Animal Experiments and Recombinant DNA Experiments of Tokyo Medical and Dental University (License number: 2010-205).

### Cells and culture

Human erythroleukaemia cell line K562 (strain RCB0027) was obtained from Riken Cell Bank, was able to synthesize globin in response to sodium butyrate, and was cultured in Ham-F12 containing 10% FCS (Invitrogen). HeLa cells were from Dr. H. Handa (Tokyo Institute of Technology) and were cultured in DMEM containing 10% FCS.

### Vectors and cloning strategy

Full length human ash1 cDNA (8892 bp) was cloned from HeLa cells by sequentially joining PCR fragments into EcoRI/XbaI sites of pCMV-tag2C (Invitrogen) to obtain pCMV-F-ASH1. Subsequently, NotI/XhoI fragment of pCMV-F-ASH1 was subcloned into EcoRI site of pCI-neo (Promega) to obtain pCI-F-ASH1. pCI-F-ASH1^1-2050^ was constructed with NotI/EcoRV fragment of pCMV-F-ASH1 by blunt end ligation into pCI-neo EcoRI site. To generate pCI-F-ASH1^1851-2694^, SalI/XhoI fragment of pCMV-F-ASH1 was subcloned into the same sites of pCMV-tag2C, from which NotI/XhoI fragment was cut out and blunt end ligated into pCI-neo EcoRI site. A histidine to lysine substitution was introduced into ASH1 at position 2113 by Site Directed Mutagenesis Kit (Stratagene) on pCI-F-ASH1. Human Hox promoter-luciferase vectors were constructed by ligating HindIII/NotI PCR fragments into the same sites of pGV-B2 (Picagene) after amplification of -600 to +186 of HoxC8 and -636 to +54 of HoxA9 promoter from BAC clones, RP11- and RP11-1134K14, respectively obtained from Dr. Inazawa (TMD, Japan). A control vector pRL-CMV, that contains the CVM promoter, was also obtained from Picagene. Expression vectors for MLL1 and MLL-AF9 were king gifts from Drs. Seto and Hess, respectively. pWHGA was a kind gift from Dr. Ptashne. Gal4DBD-ASH1 expression vectors was constructed from pGEX-6P-ASH1 vectors. Lentivirus vectors and packaging plasmids pENTR4-H1, CS-RfA-EG, pCAG-HIVgp, and pCMV-VSV-G were obtained from Riken Bioresource Center (Japan). Mouse ASH1 and MLL1 knockdown vectors were constructed by first cloning synthetic shRNA oligonucleotides into BglII and XbaI sites of pENTR4-H1 and then subcloning into CS-Raf-EG using Gateway LR Clonase (Invitrogen). Primers used of PCR cloning and mutagenesis are listed in Supplementary materials.

### Gene knockdown

Knock-down siRNA were designed by iGENE Therapeutics Inc., Japan, and double strand RNA was synthesized together with a scrambled sequence with minimal off-site effect by Hokkaido System Science (Japan). For electroporation, 1×10^5^ K562 cells were mixed with 0.5 µg siRNA in Electrolytic Buffer E and pulsed at 1200 V for 50 µsec using MP-100 (Digital Bio Tech), and then put back in Ham-F12 with 10% FCS. After 48 hours, cells were harvested and total RNA prepared with ISOGENE (Nippon Gene). Total RNA was reverse transcribed using RNA PCR Kit (AMV) Ver. 3.0 (TaKaRa-Bio, Japan) according to manufacturer's protocol. Subsequently, first strand cDNA was amplified using Power SYBR Green Master Mix and 7900HT Fast Real Time PCR System (Applied Biosystems). Relative amount of templates was given by 2^-ΔΔCT^, where ΔΔCT =  (Ct^target^ - Ct^GAPDH^)_si6925_ - (Ct^target^ - Ct^GAPDH^)_control_. Sequences of oligonucleotides are listed in Table S1.

### Chromatin immunoprecipitation

K562 and HeLa cells were fixed with 3.6% formaldehyde at room temperature, blocked with 0.2 M glycine, lysed, and sonicated using Bioraptor UCD-250 (BM Equipment Coorp., Japan). Antibodies against C-terminal end of ASH1 or internal epitope of ASH1 (Santa-Cruz) were added and immunoprecipitated with Protein G-agarose coated with sermon sperm DNA (Clontech). Cross-liking was reversed and genomic sequences quantified by real time PCR as in gene knockdown.

### Luciferase assay

HeLa cells were synchronised by double thymidine block and plated in 24-well plates at 5×10^4^ cells/well, transfected with 0.5 µg pGV-HoxC8 or pGV-HoxA9, 0.01 µg pRL-CMV, with or without 0.5 µg pCI-F-ASH1, pCXN2-MLL1, or pCI-neo to adjust total amount of plasmid using Superfect Reagent (Qiagen). After culture for 6 hours in the presence of 2 mM thymidine, cells were rinsed and incubated for further 9 hours to allow one round of cell cycle, after which 2 mM thymidine was added back to keep cells at G1/S boundary. At 48 hours after transfection, cells were lysed, protein content adjusted, and luciferase activity was measured using Dual-Luciferase Reporter Assay System (Promega) on Lumat LB9501 (Berthold).

### Bone marrow transfer and flow cytometric analysis

Haematopoietic progenitors were purified from bone marrow of C56BL/6J-Ly5.1 female mice by depleting lineage marker^+^ cells using MagCellect Mouse Haematopoietic Cell Lineage Depletion Kit (R&D Systems). Approximately 3.3×10^5^ progenitor cells were then transduced with CS-CDF-EG-PRE, CS-H1-siAsh1-EG, or CS-H1-siMll1-EG lentiviral vectors in the presence of 100 ng/ml SCF, 10 ng/ml TPO, and 10 ng/ml Flt3-L for 24 hours, and subsequently injected intravenously into five C57BL/6J-Ly5.2 recipient female mice which had been γ-irradiated by 950 cGy. After 4 weeks, mice were sacrificed and peripheral blood was analysed using CD45.1^PE^, CD45.2^biotin^, CD11b^APC^, Gr-1^APC^, CD19^Tricolour^, TCRβ^APC^, TER119^PE^ antibodies (BD Pharmingen) and streptoavidin^Tricolour^ (Caltag) on FACSCalibur (BD Bioscences).

## Supporting Information

Figure S1 **Hox code of K562 leukemic cell line.** ChIP-seq data of histone H3 trimethylation at K4, K27, and K36 and RNA polymerase II developed by Broad Institute were obtained from the ENCODE database[49]. The raw data were mapped to the human genome GRCh37/hg19 assembly using bwa[50] and peak detection was carried out using MACS[51]. Presence or absence of activation marks (K4me3, K36me3, and Pol2) and a repression mark (K27me3) indicate that subsets of HoxB and HoxC genes are transcribed in K562 cells whereas HoxA and HoxD loci are largely silent.
(TIF)

Figure S2 **ChIP analysis of the HoxC8 promoter using antibodies against the C-terminal end of ASH1.** ChIP analysis was carried out as in Fig. 1B using house-made rabbit polyclonal antibodies against the C-terminal epitope of ASH1. Preferential bindings of ASH1 to the promoter as well as downstream region of HoxC8 are shown.
(TIF)

Figure S3 **Titration of ASH1 and MLL1 in Hox gene activation.** HeLa cells were transfected with either fixed amount (1 µg) of MLL1 and different amount of ASH1 expression vectors (closed circles) or fixed amount (1 µg) of ASH1 and different amount of MLL1 (open circles) together with HoxA9-luciferase reporter. Luciferase activities corrected by CMV-renilla luciferase activities are plotted. ASH1 and MLL1 shows first order and seconder order reaction kinetics, respectively.
(TIF)

Figure S4 **Deletion mutants of ASH1.** Despite the clear evidence that full-length ASH1 has a strong transactivation potential, it is hardly detectable by Western blot analysis[7], [52]. We constructed a series of deletion mutants of ASH1 to identify fragments which can be detected and quantified by Western blot. Mutants in group (A) are not detectable and those in group (B) are detectable by Western blot. Inclusion of region III and either region I or region II of the N-terminal part of ASH1 appears to render proteins invisible by Western blot. The largest ASH1 mutant that can be identified at a protein level is ASH1^Δ216-1283^ and it has a Hox promoter activation potential comparable to that of wild type ASH1 (Fig. 2C).
(TIF)

Figure S5 **Hox promoter is sensitive to low dose okadaic acid.** HeLa cells were transfected with HoxA9-firefly luciferase (open squares) and CMV-renilla luciferase (closed squares) vectors together with ASH1 and MLL1 expression vectors (1 µg each) in the presence of absence of okadaic acid. Okadaic acid enhances transcription of HoxA9 but CMV promoter at a concentration of 1 nM which is known to inhibit protein phosphatase 2A but not protein phosphatase 1A, suggesting that Hox promoter activation by ASH1 and MLL1 is sensitive to the activity of protein phosphatase 2A.
(TIF)

Figure S6 **Effects of ASH1 and MLL1 knockdown on haematopoiesis.** Effects of ASH1 and MLL1 knockdown on haematopoietic development *in vivo*. Purified haematopoietic stem cells were transduced with lentiviral vectors expressing control GFP (closed bars) or shRNA for ASH1 (hashed bars) or MLL1(open bars) and transplanted into sublethally irradiated mice. Donor-derived cells were distinguished from host cells by Ly-5.1/Ly-5.2 allotypes, and proportions of lineage marker-positive cells among donor-derived cells are indicated. Knockdown of MLL1 caused statistically significant (p<0.05) reduction in the number of cells expressing CD11b (macrophages) or Gr-1 (granulocytes) and reciprocal increase in the number of lymphoid cells expressing CD19. The effects of ASH1 knockdown were not statistically significant but were similar to those of MLL1 knockdown.
(TIF)

Table S1 **Oligonucleotide sequences.** Sequences of oligonucleotides used for RT-PCR, ChIP-PCR, knockdown experiments, and cloning.
(DOC)

Table S2 **Hox promoter-luciferase reporter experiments.** Raw readings of firefly and renilla luciferase activities using Dual-Luciferase Reporter Assay System (Promega).
(XLS)
