# Supplementary figures and images for: Dual Function of Histone H3 Lysine 36 Methyltransferase ASH1 in Regulation of Hox Gene Expression
Source: PLoS One. 2011 Nov 28;6(11):e28171. doi: 10.1371/journal.pone.0028171 (PMC3225378; doi:10.1371/journal.pone.0028171)

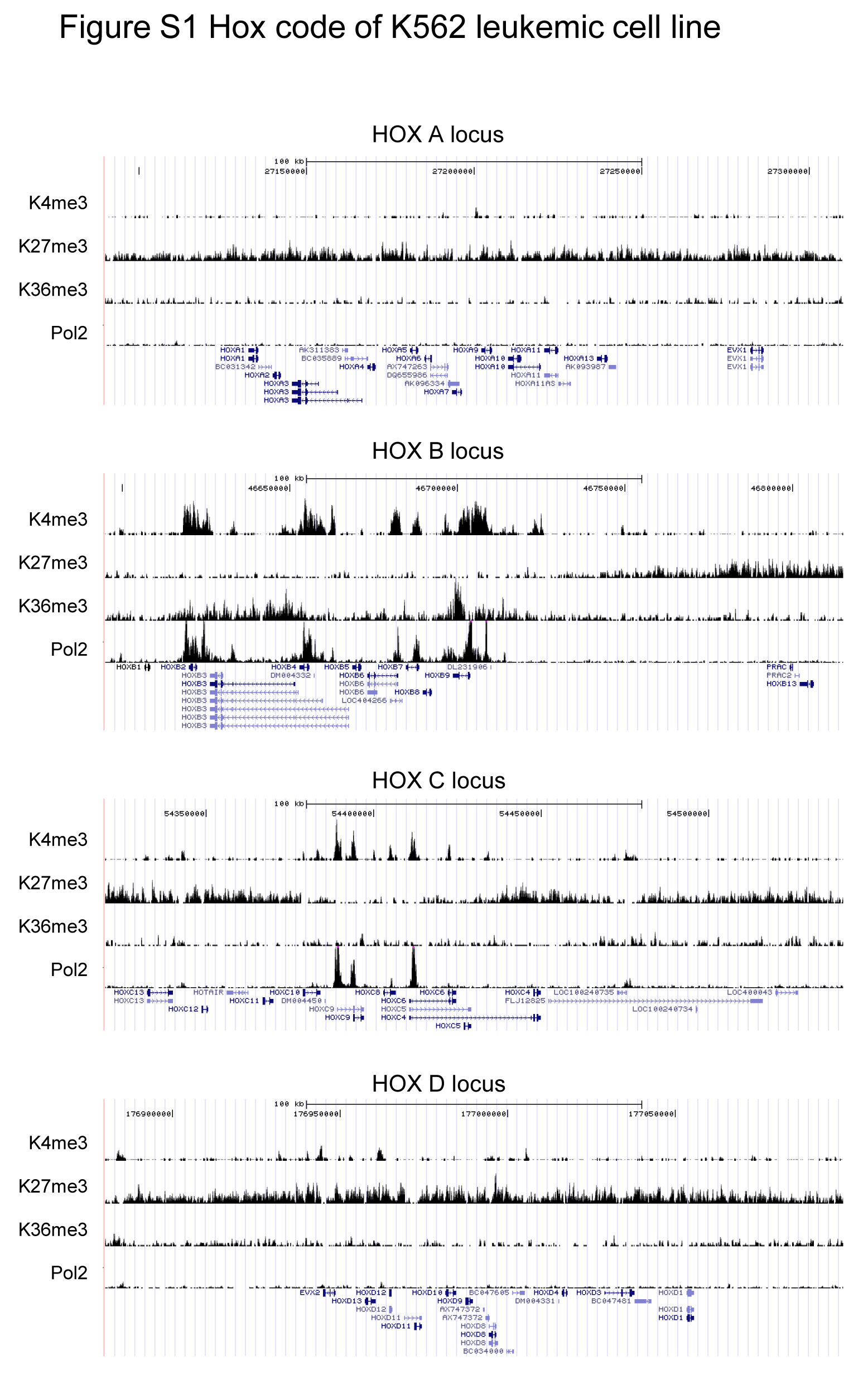

Supplement: Figure S1 — Hox code of K562 leukemic cell line. ChIP-seq data of histone H3 trimethylation at K4, K27, and K36 and RNA polymerase II developed by Broad Institute were obtained from the ENCODE database[49]. The raw data were mapped to the human genome GRCh37/hg19 assembly using bwa[50] and peak detection was carried out using MACS[51]. Presence or absence of activation marks (K4me3, K36me3, and Pol2) and a repression mark (K27me3) indicate that subsets of HoxB and HoxC genes are transcribed in K562 cells whereas HoxA and HoxD loci are largely silent. (TIF) [file pone.0028171.s001.tif]

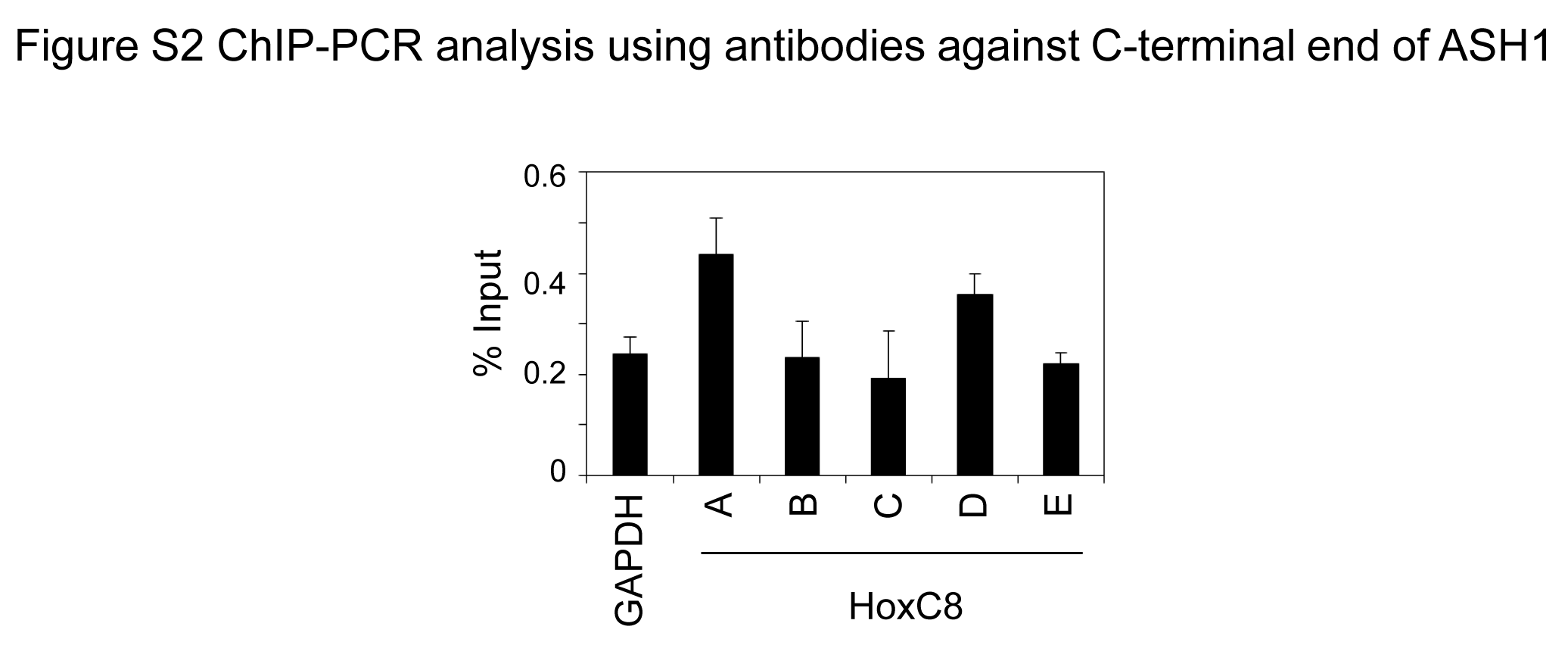

Supplement: Figure S2 — ChIP analysis of the HoxC8 promoter using antibodies against the C-terminal end of ASH1. ChIP analysis was carried out as in Fig. 1B using house-made rabbit polyclonal antibodies against the C-terminal epitope of ASH1. Preferential bindings of ASH1 to the promoter as well as downstream region of HoxC8 are shown. (TIF) [file pone.0028171.s002.tif]

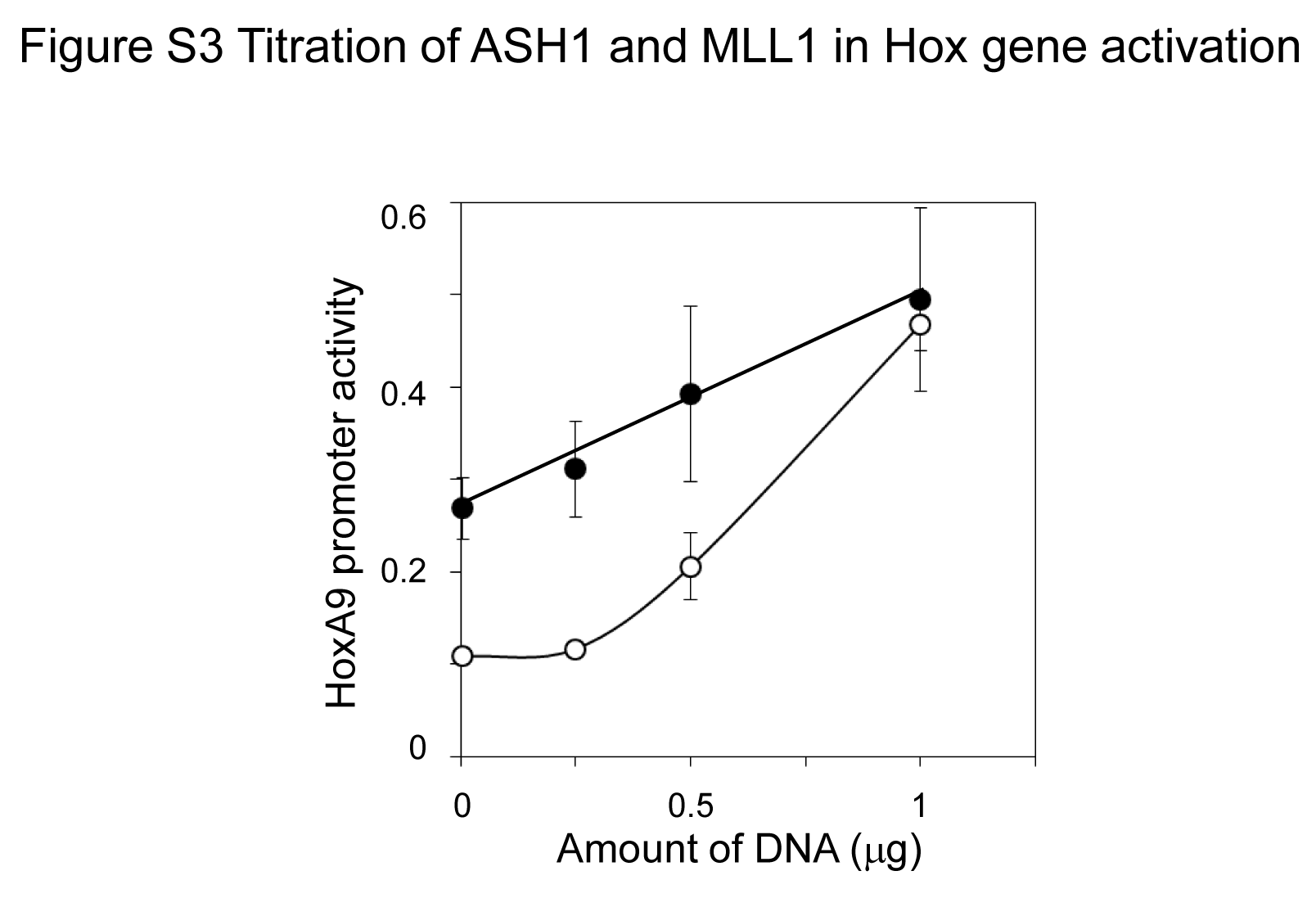

Supplement: Figure S3 — Titration of ASH1 and MLL1 in Hox gene activation. HeLa cells were transfected with either fixed amount (1 µg) of MLL1 and different amount of ASH1 expression vectors (closed circles) or fixed amount (1 µg) of ASH1 and different amount of MLL1 (open circles) together with HoxA9-luciferase reporter. Luciferase activities corrected by CMV-renilla luciferase activities are plotted. ASH1 and MLL1 shows first order and seconder order reaction kinetics, respectively. (TIF) [file pone.0028171.s003.tif]

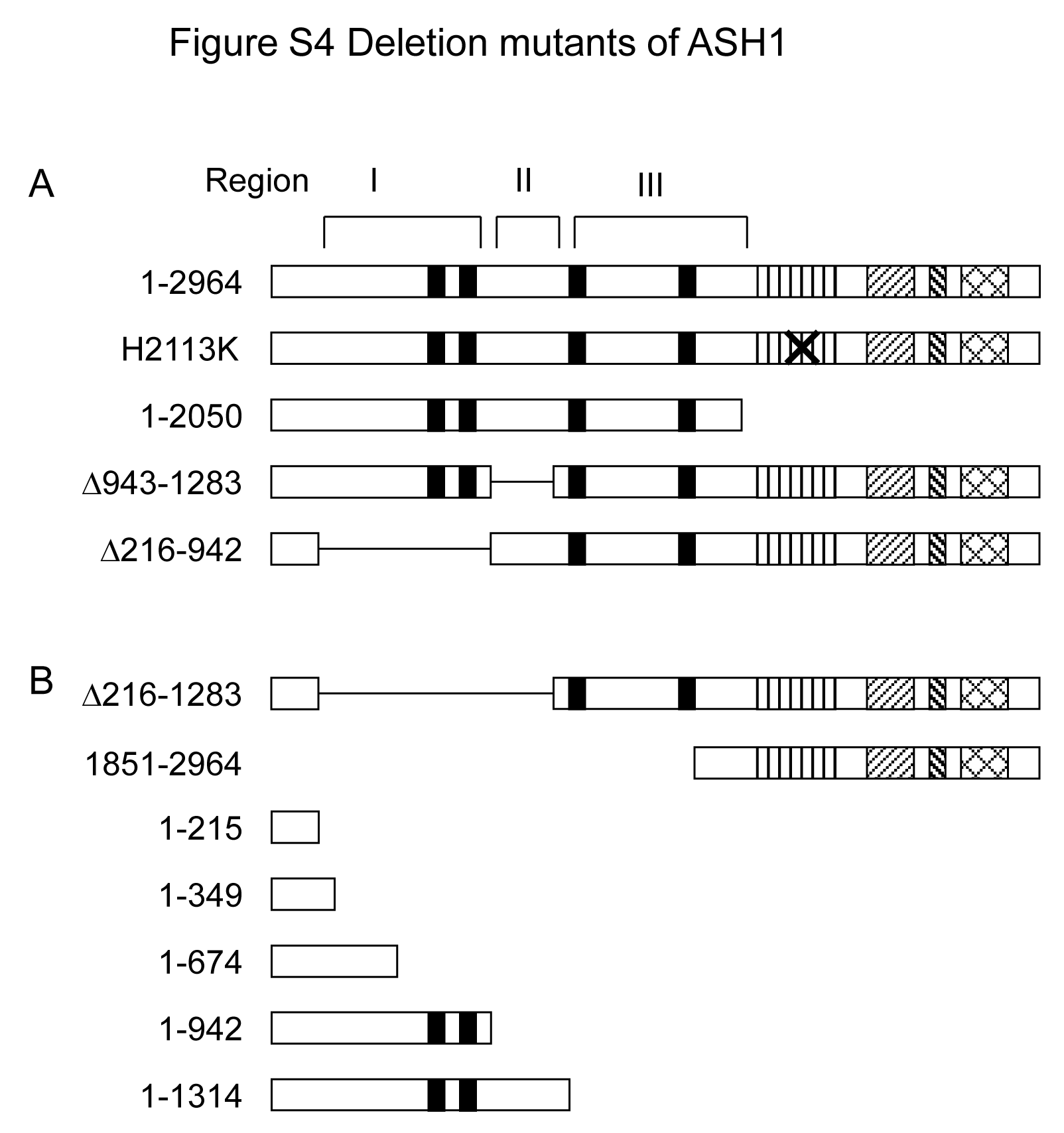

Supplement: Figure S4 — Deletion mutants of ASH1. Despite the clear evidence that full-length ASH1 has a strong transactivation potential, it is hardly detectable by Western blot analysis[7], [52]. We constructed a series of deletion mutants of ASH1 to identify fragments which can be detected and quantified by Western blot. Mutants in group (A) are not detectable and those in group (B) are detectable by Western blot. Inclusion of region III and either region I or region II of the N-terminal part of ASH1 appears to render proteins invisible by Western blot. The largest ASH1 mutant that can be identified at a protein level is ASH1Δ216-1283 and it has a Hox promoter activation potential comparable to that of wild type ASH1 (Fig. 2C). (TIF) [file pone.0028171.s004.tif]

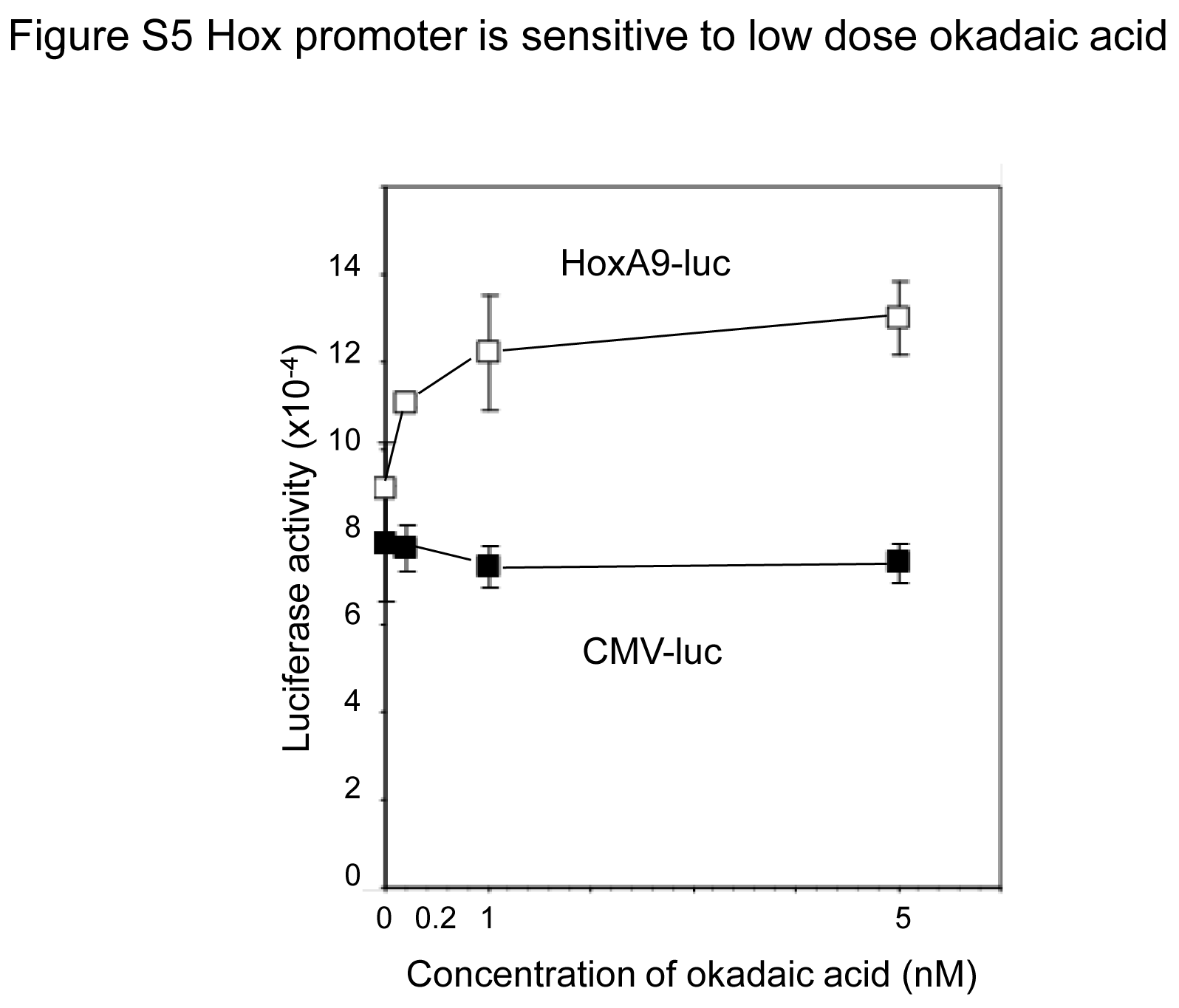

Supplement: Figure S5 — Hox promoter is sensitive to low dose okadaic acid. HeLa cells were transfected with HoxA9-firefly luciferase (open squares) and CMV-renilla luciferase (closed squares) vectors together with ASH1 and MLL1 expression vectors (1 µg each) in the presence of absence of okadaic acid. Okadaic acid enhances transcription of HoxA9 but CMV promoter at a concentration of 1 nM which is known to inhibit protein phosphatase 2A but not protein phosphatase 1A, suggesting that Hox promoter activation by ASH1 and MLL1 is sensitive to the activity of protein phosphatase 2A. (TIF) [file pone.0028171.s005.tif]

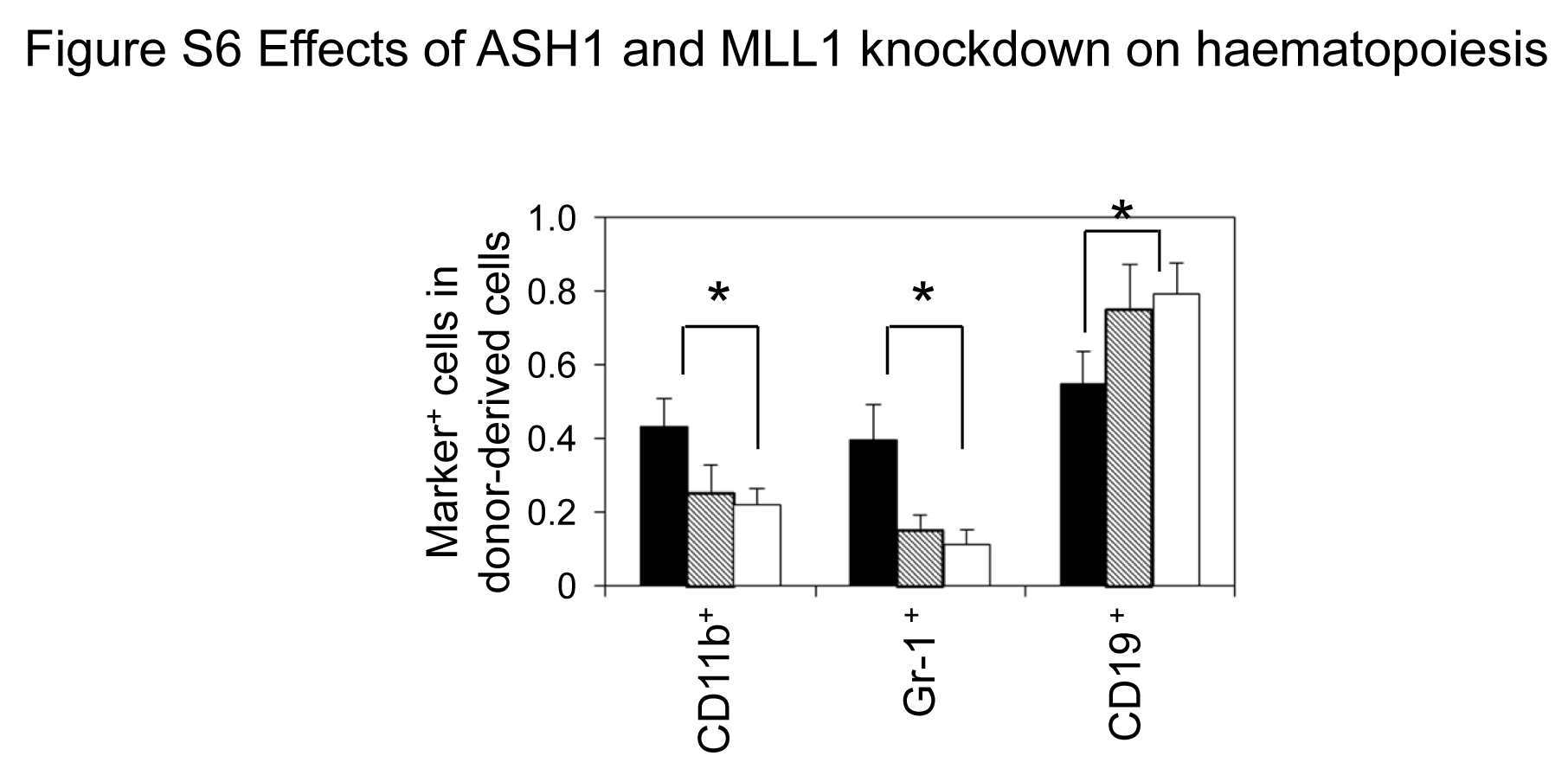

Supplement: Figure S6 — Effects of ASH1 and MLL1 knockdown on haematopoiesis. Effects of ASH1 and MLL1 knockdown on haematopoietic development in vivo. Purified haematopoietic stem cells were transduced with lentiviral vectors expressing control GFP (closed bars) or shRNA for ASH1 (hashed bars) or MLL1(open bars) and transplanted into sublethally irradiated mice. Donor-derived cells were distinguished from host cells by Ly-5.1/Ly-5.2 allotypes, and proportions of lineage marker-positive cells among donor-derived cells are indicated. Knockdown of MLL1 caused statistically significant (p<0.05) reduction in the number of cells expressing CD11b (macrophages) or Gr-1 (granulocytes) and reciprocal increase in the number of lymphoid cells expressing CD19. The effects of ASH1 knockdown were not statistically significant but were similar to those of MLL1 knockdown. (TIF) [file pone.0028171.s006.tif]
