# Supplementary material for: Dual Function of Histone H3 Lysine 36 Methyltransferase ASH1 in Regulation of Hox Gene Expression
Source: PLoS One. 2011 Nov 28;6(11):e28171. doi: 10.1371/journal.pone.0028171 (PMC3225378; doi:10.1371/journal.pone.0028171)
Supplement: Table S1 — Oligonucleotide sequences. Sequences of oligonucleotides used for RT-PCR, ChIP-PCR, knockdown experiments, and cloning. (DOC) [file pone.0028171.s007.doc]

Table S1. Oligonucleotide sequences

| Human ASH1 cloning primers | Sequence |
| --- | --- |
| Ash1-AF | GAATTCTGATGGACCCTAGAAATACTG |
| Ash1-AR | CTCGAGTACACTAGTTCCTCCATTAAG |
| Ash1-BF | ACTAGTGTAACAGAAAAGTTG |
| Ash1-BR | CTCGAGAAACCCGGGACAATACCAATTCCTAGCTTC |
| Ash1-CF | TCTAGATGTCCCGGGTTTAGTGCATAAAGAGTCT |
| Ash1-CR | AGTACTTAGGCTTTCAGCAAC |
| Ash1-DF | AGTACTAGTTTGCAGTCCAAAC |
| Ash1-DR | CTCGAGTCTGGATCCTGAAGTTGATC |
| Ash1-EF | GGATCCAGATGACCTAGATG |
| Ash1-ER | GAATTCTCCACTAGTCCCAGAAGAC |
| Ash1-FF | ACTAGTGGAGGTCAGAGCCCT |
| Ash1-FR | GAATTCTCTGGATCCTGTCTATTTCG |
| Ash1-GF | TCTAGAGACAGGATCCAGACTTTATTG |
| Ash1-GR | CTCGAGATCGGTACCGCTTCAAAGATTCCAGGAC |
| Ash1-HF | GGTACCGATTTGGAAAGGATGCTGTTGGA |
| Ash1-HR | AGTACTCTCAGATGAAGACCT |
| Ash1-IF | AGTACTTCTTCAACAGTAAAC |
| Ash1-IR | GTCGACCIGGCCTACGTT |
| Ash1-JF | GTCGACCTCGGAAATGTC |
| Ash1-JR | CTCGAGAAGGATATCATAAGGAAGCTG |
| Ash1-KF | GATATCCTTTGGCAGTGGAA |
| Ash1-KR | CTCGAGTAAGCTAGCTGAGTGAATATTATCACTG |
| Ash1-LF | GCTAGCTTATATACCCGTTGGAATGG |
| Ash1-LR | AGTACTTCTCAGCATTCCGA |
| Ash1-MF | AGTACTATGGGCGTAAATCC |
| Ash1-MR | AAGCTTCTCAATGCGAAAGA |
| Ash1-NF | AAGCTTTGGAAGAATGAAAAAG |
| Ash1-NR | CTCGAGTCACTTTCGAAAGCTGTTTTC |
| Ash1-H2113R-F | AATGAGGCCAGATTCATCAACAAAAGCTGTGACCCAAATTGTGAA |
| Ash1-H2113R-R | TTCACAATTTGGGTCACAGCTTTTGTTGATGAATCTGGCCTCATT |
|  |  |
| RT-PCR primers | Sequence |
| HoxB2F | CGAGTTCCCTTGGATGAAAG |
| HoxB3F | CAAAAGTGGTCCCCCAAAGT |
| HoxB4F | TCCCACTCCGCGTGCAAAGA |
| HoxB5F | ACCGAAATAGACGAGGCCAG |
| HoxB6F | TTCTACCGCGAGAAAGAGTC |
| HoxB7F | ACTTGGCGGCCGAGAGTAAC |
| HoxB8F | ATCCAGACCTGGTGCAGTAC |
| HoxB9F | GACAAAGAGAGGCCGGATCA |
| HoxB13F | GTCCCTTTTGGAAGGCAGCAT |
| HoxB2R | TTTCGGTGAGGTCCAGCAAG |
| HoxB3R | CCACCACAGCCCTCTGCTGT |
| HoxB4R | GATCTTGGTGTTGGGCAACT |
| HoxB5R | AGCTGTAGCCAGGCTCATAC |
| HoxB6R | AGACGCGCTGAGCAGTTTGC |
| HoxB7R | CTGCTTCAGCCCTGTCTTGG |
| HoxB8R | ATTTGCTGCTGGGGAACTTG |
| HoxB9R | AGTGAGATGGGGAAGAGCTAG |
| HoxB13R | CTGTTCTTCACCTTGGCGAG |
| HoxC4F | GCCAGCAAGCAACCCATAGT |
| HoxC5F | ACCAAACTGCACATGAGCCA |
| HoxC6F | CAGCGAATGAATTCGCACAG |
| HoxC8F | CTTTATGGGGCTCAGCAAGA |
| HoxC9F | ACAAAGAGGAGAAGGCCGAC |
| HoxC10F | AAGTGAGTTTCCCTGAGACC |
| HoxC11F | AGAACACAAATCCCAGCTCG |
| HoxC12F | GCTTGGTATCGCCGTTGAAC |
| HoxC13F | GGACAGTCAGGTGTACTGCT |
| HoxC4R | GACTTTGGTGTTGGGGAGTC |
| HoxC5R | GCTGCCTCTAAAGAGCCTCT |
| HoxC6R | TTTCCTCTTTTCCGCCCAGG |
| HoxC8R | TGTAAGTTTGCCGTCCACTG |
| HoxC9R | TTTAGGACTGCTCCTTGTCG |
| HoxC10R | CTTCCGCTCTTTGCTGTCAG |
| HoxC11R | CAGCAGAGGATTTCCCGAGA |
| HoxC12R | TTGCTCCCTCAACAGAAGTC |
| HoxC13R | AGTGGAGATGAGGCGCTTTC |
| GapdhF | TCTTCACCACCATGGAGAAG |
| GapdhR | CAGTGATGGCATGGACTGTG |
| bActF | AACCGCGAGAAGATGACCCA |
| bActR | AGGGCATACCCCTCGTAGAT |
| gGlobinF | TCTCAAGGGCACCTTTGCCCA |
| eGlobinF | AACATGGACAACCTCAAGCC |
| bGlobinF | CCTTTAGTGATGGCCTGGCT |
| gGlobinR | AGCCTATCCTTGAAAGCTCTG |
| gGlobinR | CACAGGAACACCTGCAAACT |
| gGlobinR | AGTAGTTGGACTTAGGGAAC |
| EklfF | TTGCCCTCCATCAGCACACT |
| EklfR | CTGGTCCTCAGACTTCACGT |
| Gata1F | TCCCAAGCTTCGTGGAACTC |
| Gata1R | AGTTGGTGCACTGAGTACCT |
| GPIIbF | GAGAGGGCCATTCCAATCTG |
| GPIIbR | TAGTGTAGGCTGCACCATCA |
| GPIIIaF | CCAGAGCAAAATGGGACACA |
| GPIIIaR | CCTCTGTAAACATGATGGCA |
| hAsh1-rt-F | TGAGATCATTCCCTTGGAGGCT |
| hAsh1-rt-R | TCTGGGACGTAATGAGGCGAGA |
|  |  |
| ChIP primers | Sequence |
| HoxC8-AF | TCGTGGATTGATGAACGCGA |
| HoxC8-BF | TTCGCACCACGTTCAAGACT |
| HoxC8-CF | AGCCAGAGTGTGGTGAGACT |
| HoxC8-DF | GCTTCTAACCCCAGTAGAGT |
| HoxC8-EF | TTTCCTCACTCATCCACCCT |
| HoxC8-AR | TCCTCACTGTCGGTAGGTAG |
| HoxC8-BR | TAGAATTTGGAGGCGTCTCC |
| HoxC8-CR | CTCAGGCTGGTTCTCTGAGA |
| HoxC8-DR | TCGAGCACATTGCATAAACAG |
| HoxC8-ER | GGAATCCCACTATCTCATCC |
| Gapdh-PrF | CTTGACTCCCTAGTGTCCTG |
| Gapdh-PrR | AAGGTCTTGAGGCCTGAGCT |
|  |  |
| Mouse ASH1 siRNA for cloning | Sequence |
| si2614S | GATCCCCGAGGAGGACCTAGATCCAGGGAAATGTGTGCTGTCCATTTCCTTGGATTTAGGTCTTCCTCTTTTTGGAAAT |
| si2614AS | CTAGATTTCCAAAAAGAGGAAGACCTAAATCCAAGGAAATGGACAGCACACATTTCCCTGGATCTAGGTCCTCCTCGGG |
|  |  |
| Human ASH1 siRNA for synthesis | Sequence |
| si6925 | UCUGGACGGUCAAAAGAGAAGAGAA |
|  |  |
| Mouse MLL1 siRNA for cloning | Sequence |
| mmll1-428S | GATCCCCACGAGCAGCTCTTAGGCTTACGTGTGCTGTCCGTAAACCTAAGAACTGCTCATTTTTTGGAAAT |
| mmll1-428AS | CTAGATTTCCAAAAAATGAGCAGTTCTTAGGTTTACGGACAGCACACGTAAGCCTAAGAGCTGCTCGTGGG |
|  |  |
| Human MLL1 siRNA for cloing | Sequence |
| hmll1-3utrS | GATCCCCGTGTGCCAGAGAAGATCTTACGTGTGCTGTCCGTAAGATCTTCTCTGGCACACTTTTTGGAAAT |
| hmml1-3utrAS | CTAGATTTCCAAAAAGTGTGCCAGAGAAGATCTTACGGACAGCACACGTAAGATCTTCTCTGGCACACGGG |
|  |  |
